# Fate of neutral-charged gold nanoparticles in the roots of the *Hordeum vulgare* L. cultivar Karat

**DOI:** 10.1038/s41598-017-02965-w

**Published:** 2017-06-07

**Authors:** Anna Milewska-Hendel, Maciej Zubko, Jagna Karcz, Danuta Stróż, Ewa Kurczyńska

**Affiliations:** 1 Department of Cell Biology, Faculty of Biology and Environmental Protection, University of Silesia in Katowice, 28 Jagiellońska Street, Katowice, 40-032 Poland; 2 Institute of Materials Science, Faculty of Computer Science and Materials Science, University of Silesia in Katowice, 75 Pułku Piechoty Street 1a, Chorzów, 41-500 Poland; 3 Laboratory of Scanning Electron Microscopy, Faculty of Biology and Environmental Protection, University of Silesia in Katowice, 28 Jagiellońska Street, Katowice, 40-032 Poland

## Abstract

Nanoparticles (NPs) have a significant impact on the environment and living organisms. The influence of NPs on plants is intensively studied and most of the data indicate that NPs can penetrate into plants. The studies presented here were performed on the roots of *Hordeum vulgare* L. seedlings using neutral-charge gold nanoparticles (AuNPs) of different sizes. In contrast to the majority of the published data, the results presented here showed that during the culture period, AuNPs: 1/did not enter the root regardless of their size and concentration, 2/that are applied directly into the cells of a root do not move into neighbouring cells. The results that were obtained indicate that in order to extend our knowledge about the mechanisms of the interactions between NPs and plants, further studies including, among others, on different species and a variety of growth conditions are needed.

## Introduction

Nanotechnology is a field of study that has been developing intensively in recent years due to the great expectations of its applications in various areas of life. The development of nanotechnology has increased the risk of the accumulation of nanomaterials (NMs) in the environment^1^. Evaluating the influence of NMs on terrestrial plants will assess the ecological hazards that might be caused by the transfer of these materials into the environment as well as their potential effect on human due to exposure through the food chain^2^. Widely carried out analyses have primarily been connected with the impact of nanomaterials on bacteria, animals, humans, and in recent years, knowledge about their impact on plants is also growing (for a review see refs 3–5). Studies have been focused on the influence of nanoparticles (NPs) on the physiological and biochemical processes that are associated with plant growth and development with a special emphasis on crop plants^6–10^. Despite the growing number of studies that have been undertaken to describe the influence of NPs on plant growth, we are still far from having complete knowledge on the impact of NPs on plant development. One of the missing issues is the lack of unified information. In order to fill this gap, the uptake, translocation and accumulation of NMs in plants requires further studies because, at present, the knowledge about this issue is still inconsistent and does not allow a simple conclusion to be drawn. This is probably caused by the variability in the physicochemical parameters of the nanoparticles that are used (diameter, surface charge and area, capping agents), their type and incubation parameters (time and concentration) as well as different growth conditions (e.g. solid, hydroponic or *in vitro* culture) the different plant species, organs and tissues that are used in the studies.

Gold nanoparticles (AuNPs) have attracted special interest because of their many industrial and biomedical applications, which lead to their release into the environment^11^. Literature data have shown that in the case of AuNPs not only the mechanisms of their uptake into plants are far from being determined (for review see ref. 12). Among metal-based nanoparticles, gold nanoparticles are the least examined in relation to germination, water balance, nutrition, genotoxicology or seed production for which the impact of AuNPs is unknown. The uptake or translocation of AuNPs is also poorly understood and the results are not conclusive^12^. Studies that have been conducted to data have been performed on, among others, plant such as Sinapsis, Glicine, Hordeum, Egeria, Azolla, Tabaco, Triticum and Oryza^13–21^ have indicated that the penetration of gold nanoparticles is species dependent and size selective^22^.

The results of studies on *Hordeum vulgare* L. cultivar Karat that are presented here were undertaken to contribute to increasing our knowledge about the interaction of AuNPs with crop plants, even if only to a small degree. The results that were obtained showed that in this variety of Hordeum, AuNPs regardless of their size (5 nm and 20 nm) and concentration (10 µm/ml and 50 µm/ml) or the length of the treatment (several hours and a few days) did not enter the roots in the hydroponic culture. The High-Resolution Transmission Electron Microscopy (HRTEM) technique^23^ was used to determine the physical nature of the electron-dense dots within the samples, which enabled the precise distinction between the AuNPs and the other electron-dense dots that were present in the electronograms of the root cells. Analyses were performed to verify the presence of AuNPs in the cell compartments, both in the symplast and the apoplast. The nature of the electron-dense dots on the electronograms and the determination of their physico-chemical characteristic were also confirmed using crystallographic methods. The results that were obtained not only add information about the NPs-plant interactions to our knowledge but also indicate that the response of plants can be diverse even among different cultivars of the same species.

## Results

### HRTEM verification of the physical properties of the AuNPs

**Figure 1:**
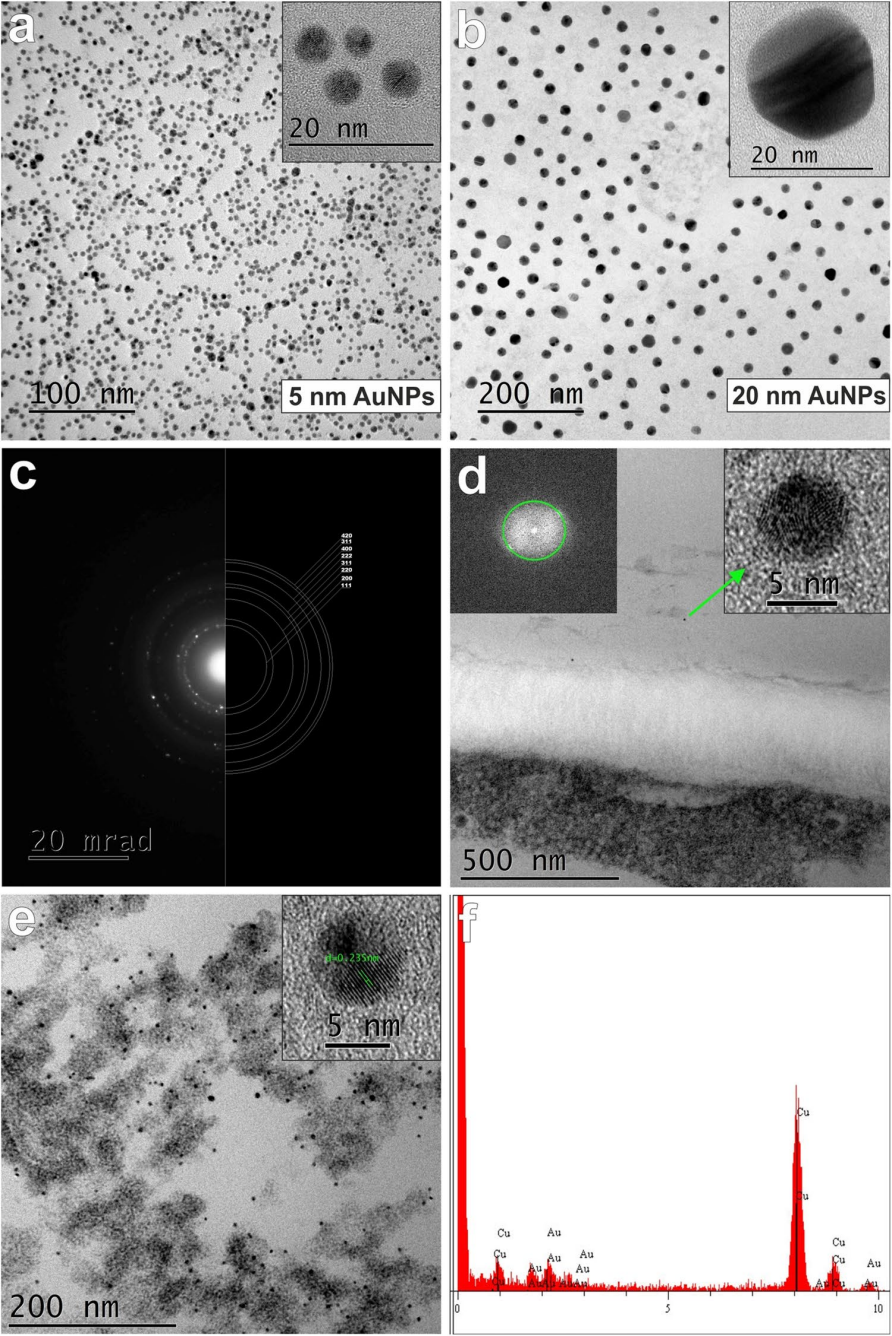
High-resolution images of the gold nanoparticles that were used in the presented studies. (**a**) 5 nm AuNPs bright field. (**b**) 20 nm AuNPs bright field. (**c**) Recorded diffraction pattern with corresponding theoretical rings for the Au structure. The diffraction pattern clearly supports the claim that the observed NPs are gold. (**d**,**e**) Evidence of the presence of AuNPs in root cells. (**d**) Single AuNP localised in the vicinity of the cell wall. HRTEM image of an AuNP with the calculated Fourier transform in the insert in the upper left-hand corner. A green circle indicates a 0.23 nm interplanar distance, which permitted the presented AuNPs to be identified. (**e**) Parenchyma cell with numerous AuNPs in the cytoplasm. The presented high-resolution images and analysis of the automatic interplanar distance indicate that the observed particles are AuNPs (the insert). (**f**) EDS-TEM analysis of the nanoparticles in the cytoplasm of the root cells. (**a,b,d,e**) Inserts in the top right-hand corners show high-resolution images of the selected AuNPs.

To confirm the size and crystal structure of the gold nanoparticles that were used in the experiments (5 nm and 20 nm), undiluted solutions of AuNPs (separate for both sizes) were placed on carbon-coated copper grids and were subjected to HRTEM analysis (Fig. 1a,b). The analyses confirmed the size as well as the crystal structure of the nanogold and suspensions that were used (Fig. 1a,b). Electron crystallography methods including diffraction, Fourier transformation and analysis of the interplanar distances of the nanogold were also conducted (Fig. 1c–e). These analyses were necessary for the further experiments, whose aims were to identify the nanoparticles in the plant material in order to answer the main question of whether gold nanoparticles penetrate into the roots. To avoid erroneous conclusions during the further analyses, all of the “electron-dense dots” in the cells were tested in order to determine whether they had the appropriate features for a gold crystal structure (Fig. 1d,e). All of the analyses that were conducted, including EDS-TEM (Fig. 1f), showed that all of the methods for detecting AuNPs were selected correctly and that there were adequate controls that allowed AuNPs to be distinguished from the other electron-dense dots that were detected in the plant sections.

### Roots of the barley seedlings that were grown in an AuNPs hydroponic solution

**Figure 2:**
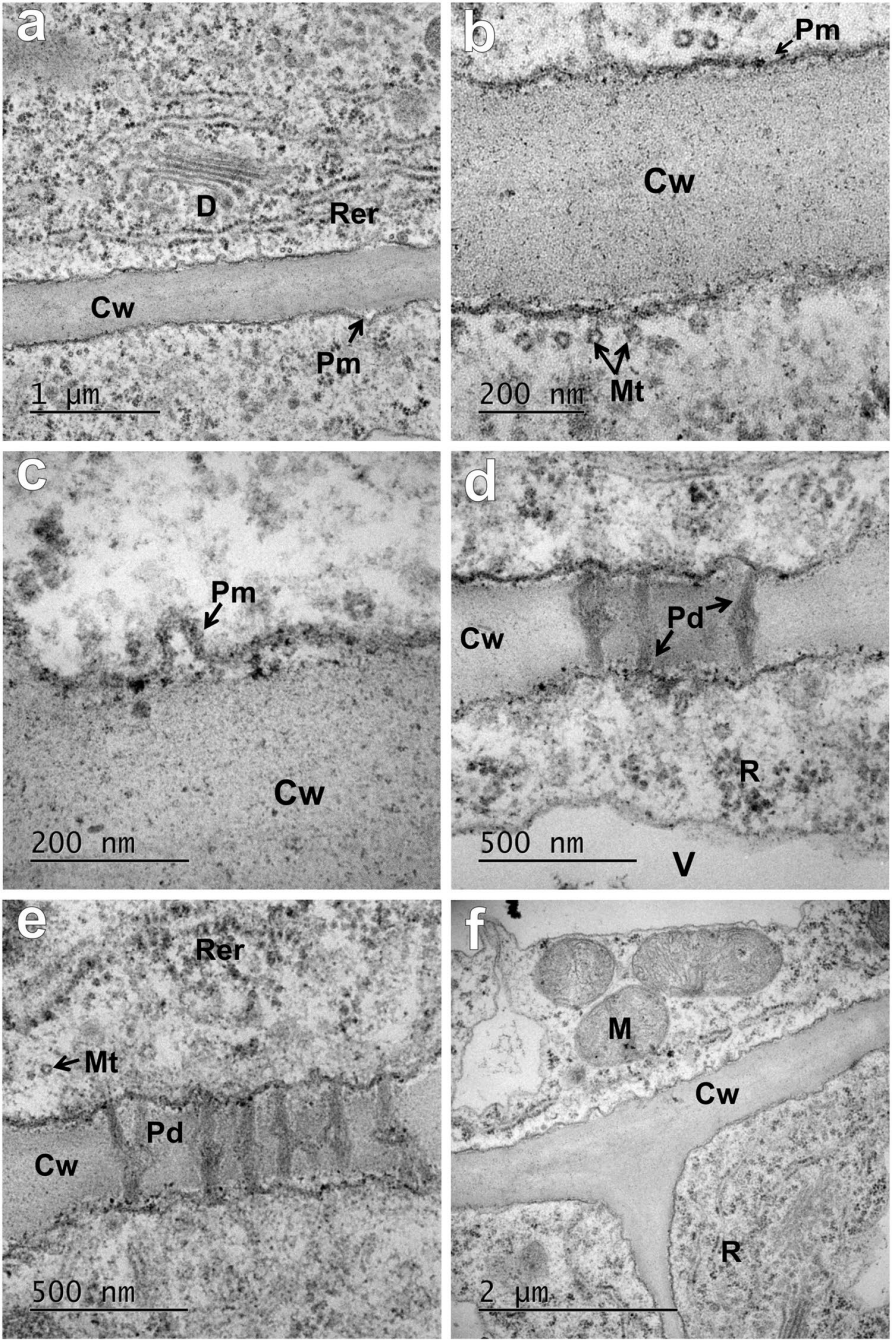
Representative ultrastructure of the cells of barley seedling roots grown in a 5 nm AuNPs hydroponic solution. (**a–e**) Parenchyma cells; (**f**) Vascular tissue; (**a–c**) Concentration of 10 µg/ml of AuNPs in the medium; (**d–f**) Concentration of 50 µg/ml of AuNPs in the medium; (**a–f**) Gold nanoparticles were not present in any of the cell organelles regardless of the type of tissue. It was confirmed that the electron-dense dots, which were considered to be nanoparticles at first, were not AuNPs as they did not show the crystal structure of an Au (staining with osmium tetraoxide, lead citrate and uranyl acetate). Cw – cell wall; D – dictyosome; M – mitochondrion; Mt – microtubule; Pd – plasmodesmata; Pm – plasmalemma; R – ribosome; Rer – rough endoplasmic reticulum; V – vacuole.

Analysis of the ultrastructure of the roots that had been exposed to 5 nm AuNPs in two different concentrations, 10 µg/ml and 50 µg/ml, showed that NPs did not penetrate into the root cells regardless of the concentration (Fig. 2a–f), and were not found in the cell wall or cytoplasm, the cell organelles or the plasmodesmata (PD; Fig. 2a–f). No NPs were found in the rhizodermis, parenchyma or vascular bundle of root regardless of the root developmental zone. The cell ultrastructure of the roots in the treated seedlings was characterised by many dictyosomes (Fig. 2a,e), abundant rough endoplasmic reticulum (Fig. 2a), microtubules in the vicinity of the cell wall (Fig. 2b,e), mitochondria and numerous ribosomes (Fig. 2f). There were no differences as compared to the samples from the control plants. The presence of numerous ribosomes and plasmalemma folding (Fig. 2b–d) indicates the intensive endo- and/or exocytosis and metabolic activity of the cells.

**Figure 3:**
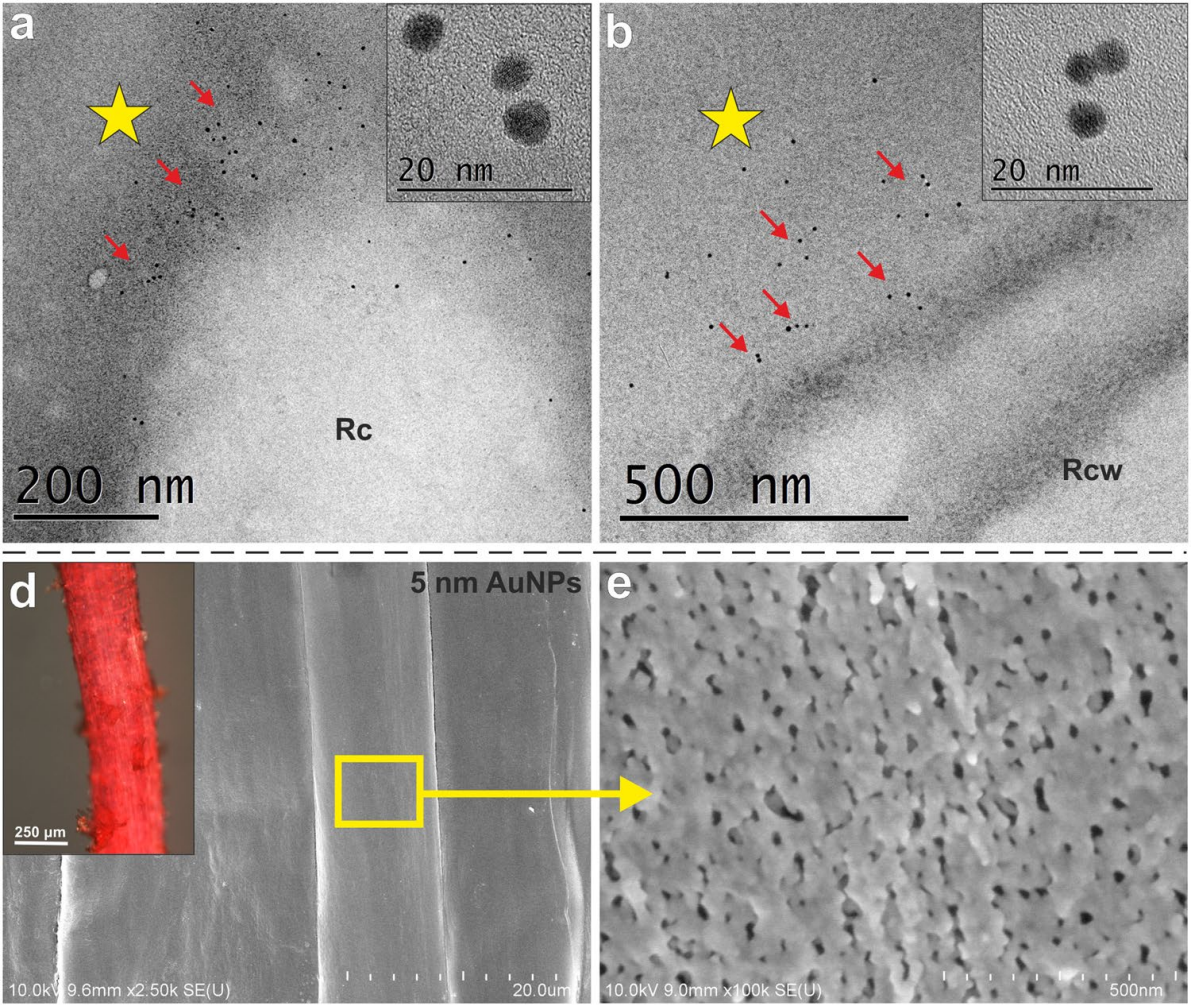
HRTEM and FE-SEM images of the rhizodermis of barley seedling roots grown in hydroponic conditions. Images obtained using the HRTEM (**a,b**) and FE-SEM (**c,d**) of barley roots grown in the 5 nm AuNPs solution (50 µg/ml) (**a–d**). (**a**) Accumulation of AuNPs (red arrows) around the root cap cell. (**b**) Accumulation of AuNPs (red arrows) on the surface of the outer periclinal cell wall of the rhizodermis. Yellow asterisks indicate mucilage at the root surface. Inserts (**a,b**) in top right-hand corners show the high-resolution images of the selected AuNPs which confirm the characteristic for the gold crystal structure. (**c,d**) SEM micrographs showing the mucilage on the root surface. (**c**) Insert in the upper left-hand corner shows stereo microscope images of barley roots stained with neutral red to detect mucilage. Rcw – rhizodermal cell wall; Rc – root cap cell.

A large number of nanoparticles were found on the surface of the root cap (Fig. 3a) and the rhizodermis (Fig. 3b) of the seedling that had been treated with nanoparticles. Although the 5 nm gold nanoparticles did not pass through the cell wall barrier, they were retained on the root surface by the mucilage. To confirm the presence of polysaccharide mucilage on the surface of barley roots, staining with neutral red was performed, and red colour of the surface indicated the positive effect of this reaction and the presence of mucilage (Fig. 3d insert). The scanning electron microscopy analysis confirmed that the rhizodermis surface of the barley roots was mucilaginous for both control and treated plants (Fig. 3d–e).

### Barley roots treated with 5 nm AuNPs that was applied directly to the root cells

Because the results described above showed that the AuNPs did not enter the root, another experiment was designed to determine whether nanoparticles translocate within the plant body after entering the barley roots cells, and if they do, *via* which pathway – symplasmic and/or apoplastic. In order to examine the distribution of the AuNPs, nanoparticles were applied into the roots by a puncture that interrupted the longitudinal continuity of the cells from the rhizodermis to the cortex above the root hair zone. The punctured sites were easily penetrated by the NPs, and therefore, the AuNPs entered directly into the cytoplasm. As a result, we were able to analyse the distribution and fate of the AuNPs.

**Figure 4:**
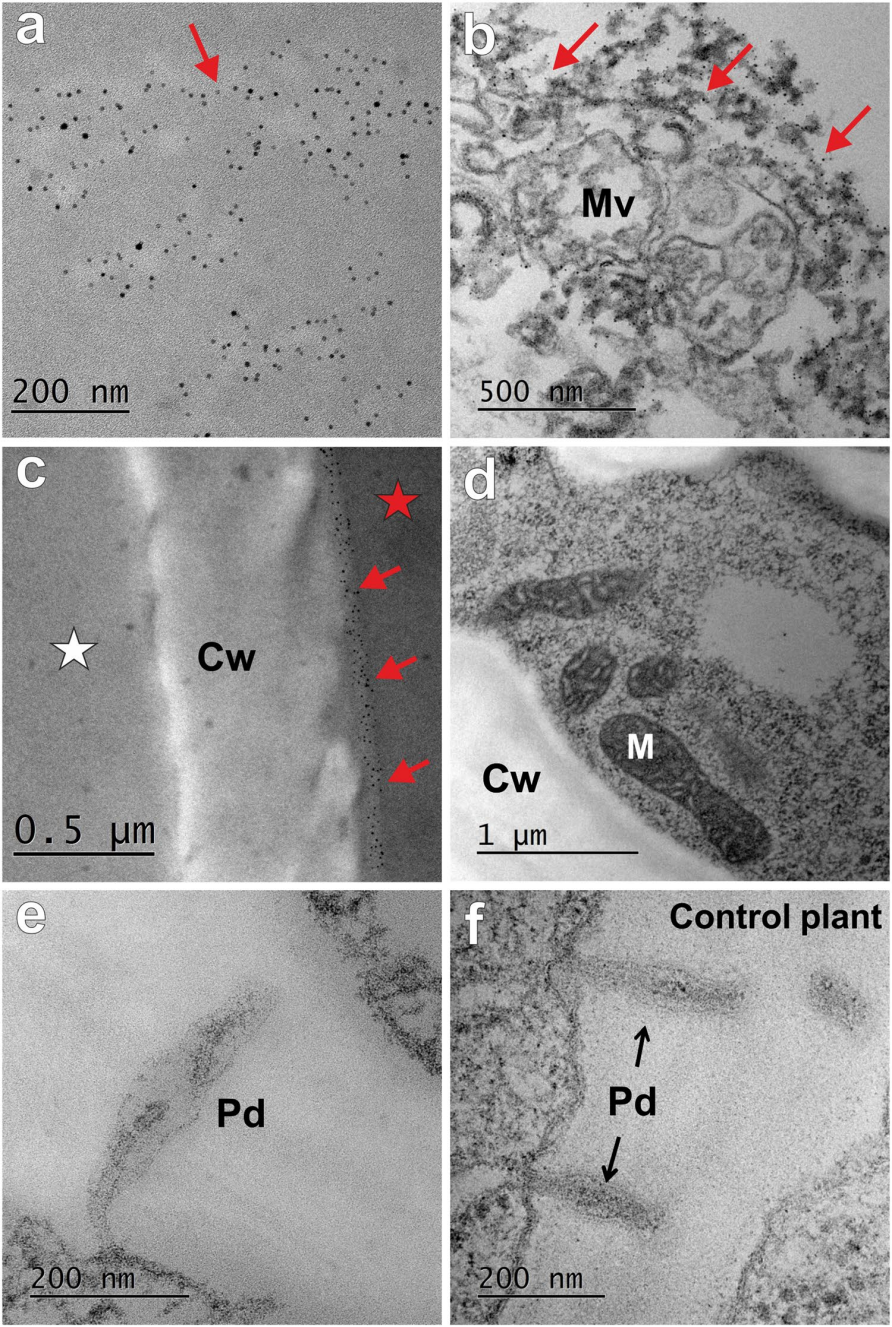
Representative ultrastructure of the punctured barley root cells that had been treated with 5 nm AuNPs (**a–e**) and a control plant (**f**). (**a**,**b**) Punctured cells with nanoparticles localised only in the cytoplasm (red arrows) but not in the organelles or plasmodesmata (**d**,**e**). (**c**) The AuNPs that were detected in the cytoplasm were observed mostly adjacent to the cell wall (red arrows) of a punctured cell (red asterisk); AuNPs were not found in neighbouring, unwounded cell (white asterisk). Cw – cell wall; M – mitochondrion; Mv – multivesicular body; Pd – plasmodesmata.

The results that were obtained showed that the nanoparticles that had been applied directly to the root parenchyma cells were only found in the cytoplasm of the punctured cells (Fig. 4a–c). No AuNPs were found in neighbouring cells (Fig. 4c, asterisk). The NPs were located in the cytoplasm that was adjacent to the wall of the punctured cells (Fig. 4c). Nanoparticles were not found in the mitochondria (Fig. 4d), vesicles of a different origin or in the multivesicular body (Fig. 4b), in the plasmodesmata (Fig. 4e) or in the cell wall (Fig. 4c–e). Cell ultrastructure into which the NPs did not pass from the punctured cells was comparable to the cells of the roots of the control plants (Fig. 4f).

### Roots of barley seedlings treated with DDG, punctured and treated with 5 nm and 20 nm AuNPs

**Figure 5:**
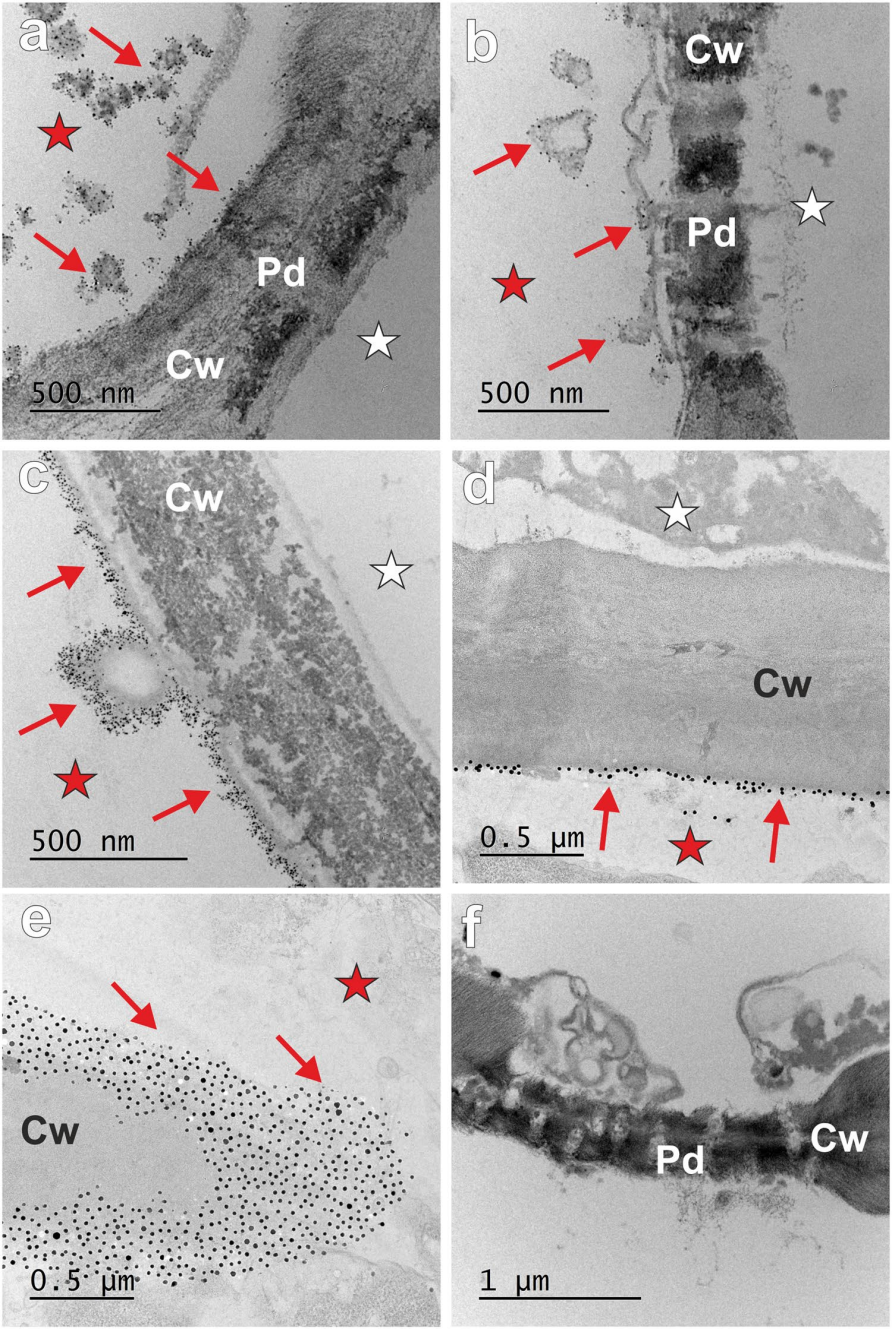
Representative ultrastructure of the barley root cells that had been incubated with DDG and treated with nanogold that was applied directly to the cell. (**a**–**c**) Roots treated with 5 nm AuNPs. (**d–f**) Roots treated with 20 nm AuNPs. Red arrows indicate the presence of AuNPs in punctured cells (red asterisks); white asterisks indicate unwounded cells adjacent to the punctured cells where AuNPs were not present. Cw – cell wall, Pd – plasmodesmata.

The wounded plants recognised stress within seconds to minutes and therefore chemical changes occurred in the wounded region^24^. A few minutes after wounding, callose is deposited in the plasmodesmata in order to provide a physical barrier between the wounded and healthy cells^25^. Because our aim was to verify whether gold nanoparticles move from cell-to-cell symplasmically, in a further experiment, barley roots were incubated with DDG (to prevent wound-induced callose deposition in the plasmodesmata) before the puncturing and treatment with the AuNPs. Analysis showed that nanoparticles of both sizes were retained only in the punctured cells (Fig. 5a–f). Although they were only observed in the cytoplasm, mostly in the vicinity of the cell wall, no AuNPs were present in the adjacent cells that had not been punctured (Fig. 5a–d, white asterisks). Furthermore, nanoparticles were absent from the plasmodesmata connecting the wounded and neighbouring cells and in the walls between those cells (Fig. 5a–d). It appeared that the treatment time had no effect on the ability of the AuNPs movement. Namely, the AuNPs that had been applied for three hours and for several days showed no symplasmic and/or apoplastic transport of NPs to the neighbouring cells.

### Determination of the pore size of the cell wall of the barley rhizodermis

The results, which showed that nanoparticles were retained on the root surface, led us to conduct the next experiment whose aim was to determine the cell wall pore diameter of the rhizodermis. Estimations of the pore size were based on the observation of the plasmolysis and cytorrhysis of the rhizodermal cells, mostly the root hair cells, of the barley roots that had been exposed to solutions of polyethylene glycol with a molecular weight that ranged from 400 Da to 10000 Da, i.e. with a diameter within the range of 2.2 nm to 7.2 nm (see Material and methods). The maximum pore size of the cell wall should correspond to the diameter of the largest polymer that is able to cause plasmolysis.

**Figure 6:**
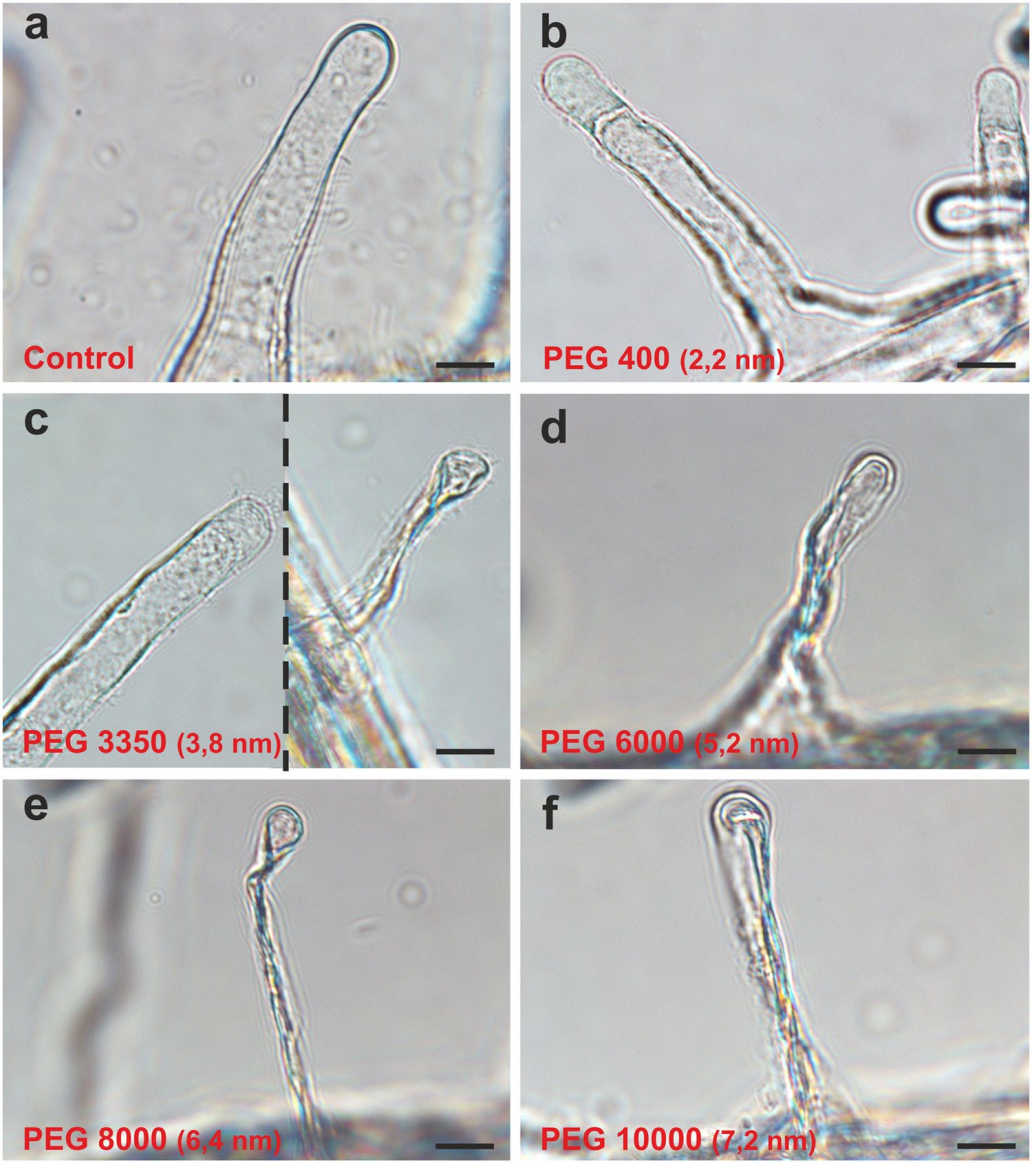
Plasmolysis and cytorrhysis that was induced in the root hairs by solutes of various molecular diameters. (**a**) The root hair before treatment (control) did not exhibit either plasmolysis or cytorrhysis. (**b**) Plasmolysed root hair cell. (**c**) Plasmolysis and cytorrhysis in the root hair cells of one root. (**d–f**) Cytorrhysis response to solutes with an increasing PEG molecular weight. Scale bars = 100 µm.

The appearance of control root hair cells is shown in Fig. 6a. A PEG 400 solution induced plasmolysis in the root hair cells (Fig. 6b). Roots that had been exposed to the PEG 3350 solution caused both plasmolysis and the collapse of the cell as a result of the cytorrhysis of the root hair cells (Fig. 6c). In the PEGs with higher molecular weights (6000 Da, 8000 Da, 10000 Da), cytorrhysis was the dominant response of the rhizodermis cells (Fig. 6d–f). Based on these results, the cell wall pore size of rhizodermis of *Hordeum vulgare* L. cv. Karat was estimated to be about 3.8 nm, which corresponds to a molecular diameter of PEG 3350.

## Discussion

Although many plants have been tested for their uptake of various kinds of nanomaterials in recent years, our knowledge is still inconsistent and many aspects remain unclear. It is extremely important to study these mechanisms in order to assess the toxicity of these materials on plants as well as to explore their possible influence and transition into the terrestrial environment and subsequently into the food chain. The aim of our work was to extend the information about the interactions between plants and nanoparticles and for this reason, we examined the interaction of gold nanoparticles and barley roots. Barley was selected because it is the most widely grown cereal (after wheat) in temperate climate zone. The results that were obtained clearly show that *Hordeum vulgare* L. cv. Karat cannot uptake 5 nm diameter PEG-coated AuNPs (what’s more, 20 nm AuNPs did not enter the root) from an aqueous medium regardless of the concentration being tested. Our results are in good agreement with the findings from an early study^26^ in which barley (cv. Proctor and Midas) was treated with colloidal, uncoated, 4.6 nm and 5.8 nm AuNPs and PVP (polyvinylpyrolidone) coated AuNPs for 41 and 22 h, respectively. The TEM analysis in that study revealed that none of the gold particles that were tested penetrated into any part of the barley roots^26^. However, the investigation of Feichtmeier *et al*.^15^ showed that barley cv. Barke did uptake 10 nm citrate-coated AuNPs from an aqueous sollution^15^. Gold nanoparticles accumulated within the roots but no translocation to the leaves was observed after a two-week exposure period^15^. Other studies on barley that had been treated with palladium nanoparticles (PdNPs) of similar sizes to those for cv. Barke exhibited both the entry of the PdNPs into the roots and their accumulation in leaves^27, 28^. However, what effect this has on plant uptake is currently unclear. Moreover, the interaction of nanoparticles with plants may not only depend on the species but also on the cultivar. Zhu *et al*.^29^ indicated that different species (*Oryza sativa*, *Lolium perenne* L., *Raphanus Dativus*, *Cucurbita mixta*) have different abilities to accumulate nanoparticles (6–10 nm AuNPs) and that it is also affected by the surface charge of the nanoparticles^29^ as has also been demonstrated by other researches^19^. Studies of the accumulation of 10-, 30- and 50 nm AuNPs that had been coated with either tannate or citrate in two different species, *Nicotiana tabacum* and *Triticum aestivum*, showed a species-dependent uptake of NPs. Tobacco accumulated AuNPs of every size and with a different surface coating, but no accumulation was observed for any treatment of wheat^18^. Furthermore, scientists^21^ revealed a size-selective uptake of AuNPs by *Nicotiana xanthi* seedlings. Small (3.5 nm) nanoparticles were taken up by the plants through the root system but 18 nm AuNPs were retained on the outer surface of the roots. These findings indicate that the penetration of nanoparticles and their movement within barley plants depend on the size and type of nanoparticles and also on their surface chemical properties. Some of the examples from the literature presented above (we apologise to all of the authors that are not mentioned here) leads to the conclusion that further studies are still needed before we fully understand the mechanisms that regulate the uptake of NPs.

The results presented here indicate that the cell wall of the rhizodermis may act as a size exclusion barrier for the entry of nanoparticles from the external environment. The sieving properties of the plant cell wall are determined by the pore size whose diameters have been determined to be a few nanometers. These were calculated to be from 3.5 nm to 3.8 nm for the root hair cells of *Raphanus sativus* and the fibres of *Gossypium hirsutum*, 3.8 nm to 4 nm for the cells of *Acer pseudoplatanus*, 4.5 nm to 5.2 nm for the isolated cells of the palisade parenchyma leaves of *Xanthium strumarium* and *Commelina communis* ^30^ and between 3.3 nm to 6.2 nm for *Chenopodium album* depending on experimental conditions^31^. Although the estimation of the wall pore size is at least partly dependent on the method that are used for such determination, it was shown that the value relies on the plant tissues, developmental and physiological state and different external/internal factors. These values indicate that particles with a diameter that is larger than the pore size would be unable to penetrate such a cell wall. Our results confirmed these assumptions and as was shown, 5 nm AuNPs were not able to pass through the cell wall of the barley rhizodermis. The results presented here showed that the cell wall pore size of the *Hordeum vulgare* L. cv. Karat rhizodermis in hydroponic is about 3.8 nm. This finding may explain why 5 nm nanoparticles were not taken up by any part of the barley roots. The results that were obtained are in contrast to some published studies. The question of whether some plants can alter the physical parameters and chemical composition of the cell wall when they interact with various types of nanoparticles and thus change the wall pores qualitatively and/or quantitatively is still open. One of the postulated mechanisms that control the uptake of exogenous substances by roots assume that the wall pore size is controlled by the cross-linking of the pectin network and by their chemical and enzymatic fragmentation, thus the diameter of the molecules that can pass through the cell wall may change^31^. It is worth mentioning here that other studies on the influence of toxic metal solutions (not nanoparticles) also showed that these metals bind to the walls of root cells, which influence the wall rigidity of the rhizodermis and leads to the rapturing rhizodermis as well as outer cortex^32^. There are still assumptions that nanoparticles may influence the root architecture^21^. Therefore, any study of the migration of nanomaterials into a plant body should take into account the size of the wall pores before and after treatment, especially when large nanoparticles penetrating into a plant are analysed.

We found that gold nanoparticles accumulate on the root surface and that they are retained by the root mucilage. This may be another reason why the AuNPs did not move from the environment into barley roots. Cucumber roots that had been exposed to 7 nm and 25 nm nanoceria also exhibited an accumulation of a large number of nanoparticles in the mucilage^2^. The root mucilage is a factor that can prevent or promote the penetration of NPs into plants. Mucilage is excreted by the surface cells of the root cap and root hairs^2, 33, 34^ and actively protects plants from different environmental stresses. However, our understanding of the interaction of nanoparticles with mucilage is still very poor. Mucilage is a highly hydrated polysaccharide that contains organic and amino acids that acidify the rhizosphere^2, 35, 36^. Therefore, the interactions of nanoparticles with mucilage depend on the physicochemical characteristics of the NPs such as their solubility, size or surface charge^29, 37, 38^.

Our analyses were also designed to verify the mobility of nanoparticles inside the plant body. The transport of NPs may be through the apoplastic and/or symplasmic pathway^37^. The cytoplasm of individual plant cells is connected by plasmodesmata, which are dynamic membranous structures that are able to control the movement of different molecules from cell to cell^36, 38^. This type of the movement of molecules is called symplasmic transport, whereas the apoplastic pathway includes the extracellular spaces outside the plasma membrane, the cell wall and intercellular spaces. To determine the movement of gold nanoparticles in barley roots, we applied AuNPs into the roots via puncturing, which allowed the NPs to penetrate directly into the cytoplasm. Regardless of whether or not we used the callose inhibitor (DDG), no movement of the AuNPs to other cells was detected. Our results strongly suggest that AuNPs do not translocate between the root cells in any direction. AuNPs were only observed in the punctured cells where they tended to accumulate in the cytoplasm that was adjacent to the wall. As no NPs were detected in the cell wall, intercellular spaces or in plasmodesmata, and what is most important, in the neighbouring cells, we postulate that nanoparticles do not move apoplastically or symplasmically in barley cultivar that was studied, at least in the culture conditions that were used here. Researches on the penetration of nanoparticles into wounded plants have shown that they can accumulate most NPs or QDs only in the first few centimetres from the injured stems^39, 40^ which indicates that the translocation of NPs is limited, at least in some plants.

The literature data showed that nanoparticles are mainly detected in the apoplast compared to the symplast^37^, thus indicating an apoplastic pathway. An accumulation of nanoparticles in the intercellular spaces and in the cell wall in roots was found in cucumber^41, 42^. An apoplastic pathway was also suggested for QD transport in maize^43^. Studies performed by Geisler-Lee *et al*.^44^ showed that in *A. thaliana* seedlings AgNPs can move *via* the cell wall but not *via* symplasmic transport because aggregates of the NPs that are found in the plasmodesmata probably exclude this pathway^44^. In lupin, wheat and maize, 20 nm mesoporous silica nanoparticles (MSNs) translocated *via* both the apoplastic and symplasmic pathways^45^. Rice plants that had been treated with 40–70 nm carbon nanoparticles also indicated that both of these systems, the apoplast and the symplast, are engaged in the movement of nanomaterials between the cells of different tissues^46^. Moreover, TEM images of woody poplar plants that had been exposed to 15, 25 and 50 nm AuNPs revealed that the main transport mechanism for the movement of nanoparticles is *via* the plasmodesmata^47^.

Although the plasmodesmata diameter was determined to be 25–50 nm, it is necessary to take into consideration that the transport channels (cytoplasmic sleeve) have a diameter of between 1.5 to 4 nm^48^. If we assume that nanoparticles move through the cytoplasm *via* diffusion, they should pass the plasmodesmata *via* the above mentioned microchannels. This means that nanoparticles that are larger than a few nanometers cannot go through plasmodesmata, at least *via* the mechanism of simple diffusion. Therefore, the lack of 5 nm AuNPs movement through the PD that was observed in this study is not surprising. The involvement of the PD in the translocation of NPs that has been described in the literature^37^ may be the result of facilitated diffusion or a molecular modification of the PD. Facilitated transport through the plasmodesmata, which has been demonstrated for many proteins, including transcription factors and viruses^49–51^, has been well described. It is extremely important to understand this mechanism if it exists in connection with NPs. Moreover, changes in the number, structure and capacity of the plasmodesmata, as well as their modifications, regulate the movement of substances between cells and change during the growth and development of plants as well as in relation to operating biotic and abiotic factors^3, 52–54^. In view of the information above, detailed analyses of the plasmodesmata and its participation in the translocation of nanoparticles within the plant body should be conducted so that the mechanism of nanoparticle movement by the plasmodesmata can be understood.

## Conclusion

To summarise, the results reported here demonstrate that in the *Hordeum vulgare* L. cultivar Karat:

1. AuNPs with a diameter of even 5 nm (±1–2 nm) did not enter the roots from the external solution.
2. The pore size of the rhizodermis cell wall was estimated to be roughly 3.8 nm.
3. AuNPs that were applied directly to the root cortex cells were retained in their cytoplasm and did not move to adjacent cells by either the symplast or the apoplast.

## Materials and Methods

### Nanoparticles characterisation

Gold (5 nm and 20 nm spheres) nanoparticles (AuNPs) were obtained from nanoComposix Europe, the Czech Republic. The surfaces of the AuNPs were modified using polyethylene glycol (PEG), which neutralises the charge and improves the stability and dispersion of the nanoparticles in a medium. The NPs diameter of the PEG coating was between 1–2 nm.

The reasons for choosing two different AuNPs diameters were as follow: 1/the size should correspond to the cell wall pore size^30^, and 2/they were significantly higher and comparable to the sizes of the NPs that have been mentioned in the literature.

### Plant material

*Hordeum vulgare* L. cultivar Karat^55^ was used to investigate the uptake of AuNPs in monocots. Caryopses were derived from the collection of the Faculty of Biology and Environmental Protection of the Department of Genetics at the University of Silesia, and were provided through the courtesy of professor Iwona Szarejko.

### Culture and treatment

The barley caryopses were processed according to a method described earlier^56^. Briefly, the surfaces of the caryopses were sterilised by immersing them in a 20% sodium hypochlorite solution for 20 minutes and subsequently washing them three times in sterilised distilled water for five minutes after which they were left in the distilled water for imbibition (24 h at room temperature). Next, the seeds were germinated in a hydroponic solution^57^ using glass tubes sealed with Parafilm (a growth chamber under conditions of a 16 h photoperiod, 20 °C and 180 μE m^−2^ s^−1^ of light). Each experiment was repeated five times and roots from at least three seedlings were investigated under the HRTEM for each experiment.

Sets and design of experiments.

1. The caryopses (and after germination - seedlings) were grown in a 1/16-strength Hoagland medium^58^ that was enriched with: a/5 nm of AuNPs at a 10 µg/ml concentration; b/5 nm of AuNPs at a 50 µg/ml concentration; after seven days (from the initiation of the culture), the roots from at least three seedlings from each experiment were harvested (at least 15 roots were taken for further analysis).
2. The caryopses (and after germination - seedlings) were grown in a 1/16-strength Hoagland solution without AuNPs; after seven days they were gently removed from the hydroponic solution, placed on moist tissue paper that had been soaked in a Hoagland solution in a chamber with an 80% relative humidity; a drop of AuNPs was applied to the surface of the root; under a stereoscopic microscope (Olympus SZH10), the root was punctured using a 0.15 mm thick acupuncture needle at a depth of the parenchyma tissue layers above the root hair zone (depth of the needle injection was determined and monitored as follow: a/the thickness of the root tissues was calculated based on the tissue cross sections that were viewed under a light microscope (Nikon Eclipse Ni-U) and measured as the length from the rhizodermis to the vascular bundle; b/this distance was marked on the surface of the needle; under a stereomicroscope, the root was punctured by hand with the needle). 5 nm AuNPs were used at a 50 µg/ml concentration. The roots of seedlings were treated in this manner for three hours and during this period were kept in the humidified chamber. This experiment was performed in order to determine whether AuNPs that are applied directly to the root cells are able to relocate from the place of their application to neighbouring cells.
3. Because injuries can cause the deposition of callose in the plasmodesmata, which can block the movement of NPs, another set of experiment was performed. The roots of seedlings (seven days after the beginning of the culture) were treated with 0.1 mM -2deoxy-D-glucose (DDG) for 1 h to prevent the wound-induced formation of a callose^59^; the seedlings were gently removed from the hydroponic solution, placed on moist tissue paper soaked in a solution of Hogland in a chamber with an 80% relative humidity and the roots were punctured by a 0.15 mm thick acupuncture needle (as described above); after this seedlings were gently returned to the glass tubes in hydroponic conditions with 5 nm and 20 nm nanogold solution at concentration 50 µg/ml for further growth (for next five days). This experiment was performed in order to determine whether the prevention of callose deposition and the length of the exposure to NPs changed the results that had been obtained earlier.
4. The control plants were cultured in the same conditions (for each of above-mentioned sets of experiments) but without the addition of AuNPs.

### Transmission electron microscopy sample preparation

The material for the Transmission Electron Microscopy (TEM) was prepared according to Kozieradzka-Kiszkurno *et al*.^60^ with some modifications. Barley roots were fixed in 2.5% glutaraldehyde and 2.5% paraformaldehyde in a 0.05 M cacodylate buffer (Sigma) (pH 7.2) and kept for 24 h at 4 °C. Some of the plant material was postfixed in 1% osmium tetraoxide (Serva) in distilled water at 4 °C overnight, whereas the other parts were not secondarily fixed with OsO_4_. Then, the tissues were dehydrated in a graded series of ethanol and gradually embedded in Epon resin (Polysciences). The Epon was polymerised for 24 h at 60 °C. For the TEM analysis, ultrathin sections, 70 nm thick, were obtained using a Leica EM UC6 ultramicrotome and were collected on carbon-coated copper grids (300 mesh, Electron Microscopy Science). Some of the grids with sections were stained with a saturated solution of uranyl acetate (Polysciences) in 50% ethanol for 15 min and 0.04% lead citrate agents (Sigma) for 10 min. The samples were analysed using a Jeol JEM-3010 (300 kV) High Resolution Electron Microscope (HRTEM) equipped with an EDS (Energy Dispersive Spectrometry) spectrometer and a Gatan 2k × 2k Orius TM 833 SC200D CCD camera. The EDS-TEM technique for detecting nanogold was used without any limitation for the 20 nm AuNPs. A single nanoparticle could not be used for the 5 nm AuNPs, and therefore analyses were only performed for areas within a section in which a larger number of 5 AuNPs were present.

The TEM images were acquired for at least three root samples from three separate experiments.

### Scanning electron microscopy (SEM)

Samples for the SEM analysis were prepared according to the procedure that had been developed for barley roots^61^. In brief, barley roots from seven-day-old seedlings that had been grown in a hydroponic culture in a 1/16 Hoagland medium (control plants) and in a 5 nm AuNPs solution (50 µg/ml concentration) were cut into 1 cm segments and fixed in 3% glutaraldehyde in a 0.1 M sodium phosphate buffer, pH 7.2 (24 h at RT). Next, the samples were washed three times for 15 min each with a phosphate buffer followed by post-fixation in 2% osmium tetraoxide for 2 h at RT. The material was subsequently washed three times in the same buffer and dehydrated in a graded ethanol/water series of 50%, 60%, 70%, 80%, 90%, 95% and 100% (10 min each step). Next, the samples were critical-point dried using carbon dioxide in a Pelco CPD2 apparatus (Ted Pella Inc., Redding, CA, USA) and then mounted on aluminium stubs with double-sided carbon tape and sputter coated with a thin film of gold in a Pelco SC-6 sputter coater (Ted Pella Inc., Redding, CA, USA). The samples were imaged using a Hitachi SU8010 field emission scanning electron microscope FE-SEM (Hitachi High-Technologies Corporation, Tokyo, Japan).

### Determination of the pore size

The solute exclusion technique was used to estimate the pore size of the barley rhizodermis^30^. The solutes that were used to determine the pore size were polyethylene glycol (PEG) 400, 3350, 6000, 8000, and 10000 (Da) (all were obtained commercially) with molecular diameters of 2.2 nm, 3.8 nm, 5.2 nm, 6.4 nm and 7.2 nm, respectively. All of the compounds were dissolved in a 1/16 Hoagland solution and the final concentration was 0.1 M. First, the roots were mounted in a drop of water on a microscope slide (control) and next, in a drop of the solution and then they were covered with a cover slip. Observations were performed after 3 h using a Olympus BX41 microscope. Images were acquired using a digital CCD camera (Olympus XC50).

### Additional staining

Roots were stained with neutral red for detecting the polysaccharide/mucilage according to the method described by O’Brien and McCully^62^.
